# Monitoring Conformational Changes in the NDM-1 Metallo-β-lactamase by ^19^F NMR Spectroscopy**

**DOI:** 10.1002/anie.201310866

**Published:** 2014-02-24

**Authors:** Anna M Rydzik, Jürgen Brem, Sander S van Berkel, Inga Pfeffer, Anne Makena, Timothy D W Claridge, Christopher J Schofield

**Affiliations:** Department of Chemistry, University of Oxford, Chemistry Research Laboratory12 Mansfield Road, Oxford OX1 3TA (UK)

**Keywords:** ^19^F-NMR, antibiotic resistance, captopril, metallo-β-lactamases, NDM-1

## Abstract

The New Delhi metallo-β-lactamase (NDM-1) is involved in the emerging antibiotic resistance problem. Development of metallo-β-lactamases (MBLs) inhibitors has proven challenging, due to their conformational flexibility. Here we report site-selective labeling of NDM-1 with 1,1,1-trifluoro-3-bromo acetone (BFA), and its use to study binding events and conformational changes upon ligand–metal binding using ^19^F NMR spectroscopy. The results demonstrate different modes of binding of known NDM-1 inhibitors, including *l*- and *D*-captopril by monitoring the changing chemical environment of the active-site loop of NDM-1. The method described will be applicable to other MBLs and more generally to monitoring ligand-induced conformational changes.

Most clinically used antibiotics contain a β-lactam ring which is critical for the inhibition of transpeptidases involved in cell-wall biosynthesis. Bacteria have evolved mechanisms of β-lactam resistance, which often employ β-lactamase-mediated antibiotic hydrolysis.[1] The increasing resistance problem is a major public health concern,[2] rendering the development of new approaches important.[3] β-Lactamases are subdivided into serine- and metallo-β-lactamases (SBLs and MBLs).[4] Once of little clinical relevance, MBLs now threaten almost all β-lactam antibiotics.[1c] Due to variations in MBL structures, including conformational changes involving active-site proximate loops, the identification of useful MBL inhibitors is challenging.[5] MBL inhibitor discovery is hampered by the lack of knowledge about MBL solution dynamics, in particular regarding conformational changes induced by inhibitor binding. NDM-1 is a “flagship” MBL which enables resistance to new generation β-lactams; Gram-negative bacteria carrying the NDM-1 gene are often referred to as “super bugs”.[6] An enhanced mechanistic, structural and conformational understanding of NDM-1 is required for inhibitor development.

There is a general need for efficient solution-based protein-observe methods that enable determination of not only ligand affinities, but which also provide information on possible binding modes, including conformational changes. The absence of endogenous fluorine in most biological material makes ^19^F NMR spectroscopy a good method for studying biological samples.[7] ^19^F NMR spectroscopy is also an attractive approach for studying protein structure and dynamics, because ^19^F chemical shifts are sensitive to changes in local conformational environment, allowing identification of even small perturbations in local electrostatic fields.[7c, 8]

Movements in loops flanking MBL active sites are proposed to be important in substrate/inhibitor binding.[9] We envisaged that ^19^F NMR spectroscopy may be useful for monitoring ligand binding-induced changes in MBLs. To introduce a ^19^F label into NDM-1 we selected the L1 loop (residues 65–73, which links-β-strands β2 and β3, Figure S1 in the Supporting Information), because crystallographic analyses implies that the L1 loop may adopt different conformations that are dependent on ligand binding (Figure S2). The nucleophilicity of thiols coupled to the apparent absence of exposed Cys side chains in NDM-1 (which contains only one cysteine, which sits at its active site), suggested that thiol modification may be suitable for ^19^F incorporation. We produced an NDM-1 variant, substituted in the L1 loop (M67C) using an optimized expression system (see Figure S3).

We found that an ^19^F label could be efficiently introduced into NDM-1 using 3-bromo-1,1,1-trifluoroacetone (BFA).[10] The M67C NDM-1 variant was site-selectively modified by treatment with BFA, under mild conditions (5 min, phosphate buffer pH 7.0, room temperature) (Figure 1) to give a single SCH_2_(CO)CF_3_ adduct (NDM-1*) as shown by intact protein mass spectrometry (MS) analysis (Figure S4); subsequent trypsin digestion and MS fragmentation studies indentified the sole detected site of modification as Cys 67 (Figure S5). Alkylation of the active-site Cys 208 was not observed, revealing selectivity in the BFA labeling procedure. We then investigated the kinetic properties of NDM-1* compared to unlabeled NDM-1. It was found that introduction of BFA label did not alter significantly substrate affinity (i.e. similar *K*_M_ values were obtained for meropenem and nitrocefin with NDM-1* and NDM-1; Table 1 and Figure S6); further, inhibition by two representative thiols (i.e. l- and D-captopril)[11] remained of similar magnitude for both labeled and unlabeled NDM-1 variants (Table 1 and Figure S7). A decrease in the *k*_cat_ value for meropenem, but not for nitrocefin was observed, possibly reflecting specific interactions with the modified residue for the intermediates derived from the former.[9b, 11] Together with earlier studies on protein alkylation by BFA,[10,12] these results demonstrate that BFA is a useful reagent for the introduction of a ^19^F label into proteins by post-translational cysteine alkylation.

**Figure 1:**
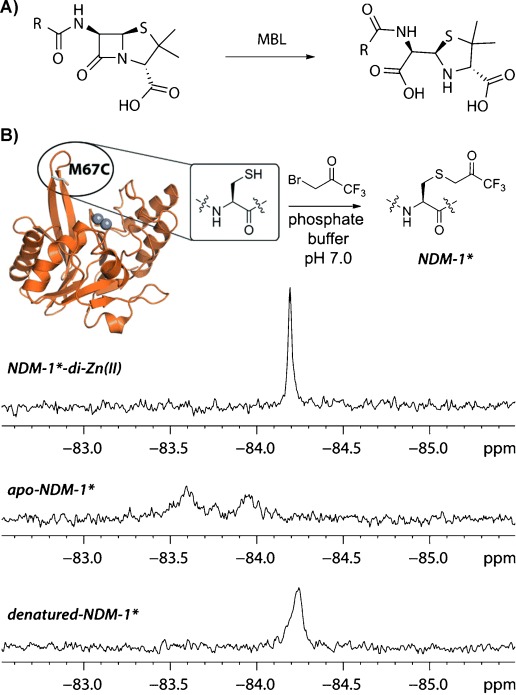
A) MBL-catalyzed β-lactam hydrolysis. B) Site-specific labeling of M67C NDM-1 with 3-bromo-1,1,1-trifluoroacetone (BFA) to give NDM-1*. ^19^F NMR spectra of NDM-1*-di-Zn^II^ complex, apo-NDM-1*, and denatured NDM-1* (obtained by incubation with 2 M guanidinium chloride) revealed distinctive signal pattern.

**Table 1:** Comparison of inhibition and kinetic properties of labeled and unlabeled NDM-1 variants.

Substrate affinity			
Enzyme	Substrate	*K*_M_ [μM]	*k*_cat_ [s^−1^]
NDM-1	Meropenem	76.6±4.4	235.2±5.6
NDM-1^*^	Meropenem	54.8±9.4	67.8±4.3
NDM-1	Nitrocefin	8.8±2.3^[a]^	25.3±1.6^[a]^
NDM-1^*^	Nitrocefin	6.2±0.7	22.9±0.6

Inhibition			
Enzyme	Inhibitor	IC_50_ [μM]	*K*_D_ [μM]^[b]^
NDM-1	l-captopril	9.4±1.4	–
NDM-1^*^	l-captopril	12.5±1.4	16.9±3.5
NDM-1	D-captopril	2.2±0.9	–
NDM-1^*^	D-captopril	2.9±1.4	5.5±2.3

[a] Data from Ref. [20]. [b] Measured by ^19^F NMR assay.

Several conformations of the L1 loop in the apo, metal and/or ligand bound states of NDM-1 have been observed crystallographically, and the L1 loop is proposed to participate in the positioning of active-site ligands (Figure S2).[9a,b] The ^19^F NMR spectrum of di-Zn^II^ bound NDM-1* displays a single protein-derived peak, suggesting that di-Zn^II^ NDM-1* exists, at least predominantly, in a single distinct conformation or that loop movement is fast relative to the NMR shift timescale[13] (Figure 1).

To investigate the local environment of the ^19^F label within the protein structure, we investigated its solvent accessibility. ^19^F NMR chemical shifts are highly sensitive to local chemical environment and even small differences in dielectric properties of a solvent, for example, change from H_2_O to D_2_O, can result in a measurable change in chemical shift. This change is proportional to the molar fraction of D_2_O in H_2_O, and extent of that change is dependent upon the accessibility of fluorine-containing moiety to solvent molecules.[8b, 14] The results of measurements of chemical shift change of NDM-1* label as function of D_2_O content using CF_3_COOH (TFA) as an internal standard reveals the ^19^F label is only slightly less sensitive to solvent interactions than TFA (85 % solvent accessibility relative to TFA) (Figure S8A), indicating that the modified Met67 residue is largely solvent exposed in the unligated NDM-1 active site.

Removal of both Zn^II^ ions from NDM-1* using 10 mM EDTA (ethylenediamine tetraacetate), led to the observation of broader signals reflecting at least two conformers of apo-NDM-1* in a intermediate exchange system (exchange rates are considered relative to NMR chemical shift time-scales[13] throughout), as indicated by significant line broadening (Figure 1). Metal sequestration from the active site was noticeably slower in the presence of the NDM-1 inhibitor D-captopril, which chelates both active site zinc ions via its thiol moiety (Figure S9).[15] Upon denaturation of NDM-1* di-zinc, using 2 M guanidinium chloride, a substantially broadened signal was obtained (Figure 1). The change in signal shape likely reflects the average chemical environment of the label in unfolded NDM-1*. These initial studies reveal the utility of ^19^F NMR spectroscopy for time-resolved monitoring of ligand induced conformational changes of NDM-1. To investigate whether conformational changes upon ligand binding could be monitored by ^19^F NMR spectroscopy we then carried out assays with di-Zn^II^-NDM-1* and different ligands.

Both l-(*S*,*S*)- and D-(*S*,*R*)-captopril are NDM-1 inhibitors (with reported IC_50_ of 202 μM and 7.9 μM,[15] and *K*_i_ of 3.9 and 1.3 μM[11] for l- and D-captopril, respectively). Titration of NDM-1* with l- and D-captopril, followed by ^19^F NMR spectroscopy, provides a direct read-out of ligand–enzyme complex formation (Figure S10). For l- and D-captopril, the appearance of distinct peaks corresponding to the di-Zn^II^-NDM-1* inhibitor complex were observed, the areas of which increases upon ligand titration (Figure 2 A). This scenario is characteristic of a slow exchange system in which both l- and D-captopril are relatively strong binders. ^19^F NMR titration data were used to calculate *K*_D_ values (Figure S11), using a modified version of a reported function.[16] To calculate the molar fraction of protein–ligand complex, Equation (1) was used:

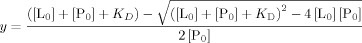
(1)

**Figure 2:**
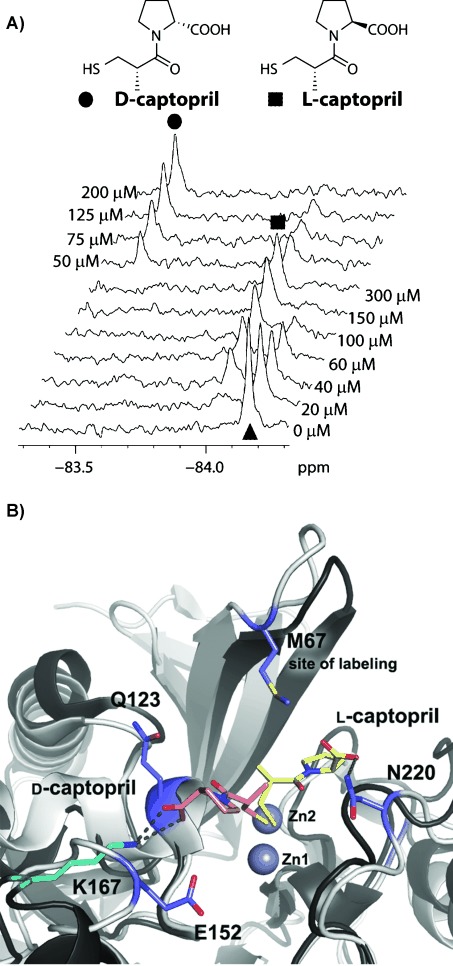
Monitoring of inhibitor binding to NDM-1 MBL by ^19^F NMR spectroscopy. A) Titration of NDM-1*-di-Zn^II^ (▴) with captopril stereoisomers leads to the appearance of distinctive peaks attributed to l- (▪) and D-captopril (•) complexes. l-Captopril can be displaced from a NDM-1* by D-captopril. B) The different chemical shifts for l- and D-captopril likely reflect different binding modes. View from crystallographic analyses of NDM-1 complexed with di-Zn^II^ and l-captopril (PDB id: 4EXS). The l-captopril (yellow) interacts with Asn220. Binding of D-captopril (pink) is modeled based on a structure of D-captopril complexed with the BlaB MBL (PDB id: 1M2X).

where [P_0_] is the total protein concentration and [L_0_] is the total ligand concentration titrated into solution. The obtained *K*_D_ values of 5.5 μM and 16.9 μM for D- and l-captopril, respectively, are in good correlation with the measured IC_50_ values (Table 1).

^19^F NMR measurements were applied to study competition between the binding of l- and D-captopril. A rise in peak intensity of the signal assigned to the D-captopril-NDM-1* complex was observed when l-captopril-NDM-1* complex was titrated with D-captopril (Figure 2 A), consistent with the lower observed IC_50_ for D- compared to l-captopril, and demonstrating that the ^19^F NMR spectra can discriminate between different binding modes.

The relatively different ^19^F chemical shifts of D- versus l-captopril suggests that the two stereoisomers bind differently. A crystal structure of NDM-1 in complex with l-captopril reveals that its thiol is positioned to chelate both active-site Zn^II^ ions and that its side-chain carboxylate interacts with Asn 220 of NDM-1 (Figure 2 B).[11] The precise binding mode of D-captopril to NDM-1 is unknown; however, it is likely that its thiol interacts with two Zn^II^ ions in the active site analogously to l-captopril.[17] We propose that the carboxylate of D-captopril likely interacts with other conserved residues, that is, Glu 123 and/or Gln 152 (Figure 2 B), analogously to the way D-captopril binds to another metallo-β-lactamase, BlaB, as observed crystallographically (Figure 2 B).[18] The observed change in the ^19^F chemical shift upon titration of di-Zn^II^-NDM-1* is much larger for D-captopril than for l-captopril indicating a more significant change of the environment upon binding of D-captopril (Figure 2 A). Analysis of available structures together with the predicted D-captopril binding mode (Figure 2 B) suggests that this difference may reflect more substantial ligand-binding induced movement of the L1 loop in the case of D-captopril. In support of this proposal, solvent exposure analyses with D-captopril show the di-Zn^II^-NDM1*-D-captopril complex has a decreased exposure of 73 % relative to the TFA response, as compared to 85 % for the di-Zn^II^-NDM1* (Figure S8B), consistent with induced loop closure on D-captopril binding. In contrast no change of solvent exposure for di-Zn^II^-NDM-1*-l-captopril complex compared to di-Zn^II^-NDM-1* was observed (Figure S8C).

To test of the generality of the ^19^F-method, we screened other inhibitors, that is, thiol-containing benzoic acids thiosalicylic acid (**1**) and 3,5-bis(mercaptomethyl)benzoic acid (**2**), which are reported to be relatively broad-spectrum MBL inhibitors.[19] Unlike the captopril isomers, upon binding of both compound **1** and **2** two new peaks were observed in the ^19^F NMR spectra (Figure 3 A). These likely arise from the binding of both **1** and **2** in two different conformations. However, the greater change in chemical shift in one of the di-Zn^II^-NDM-1*-**1** complexes suggests that the phenyl ring of **1** may interact more directly with labeled L1 loop. In the case of thiol **2**, two binding modes were also observed, with one significantly deshielded (Figure 3 A). The latter species may correspond to a significant conformational change of the flexible L1 loop (as indicated by the larger change in the chemical shift) (Figure 3 A).

**Figure 3:**
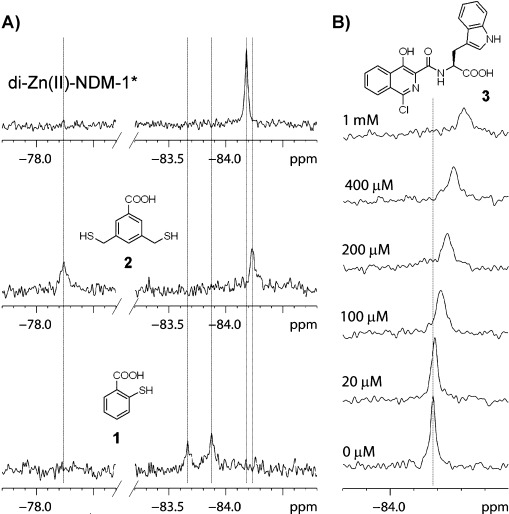
^19^F NMR spectroscopy enables identification of different modes of ligand binding. A) Titration of di-Zn^II^-NDM-1* (70 μM) with thiols 1 and 2 (160 μM) implies different binding modes. B) Titration of di-Zn^II^-NDM-1* (70 μM) with isoquinoline 3; averaged signal of di-Zn^II^-NDM-1* non-bound and in complex with 3 indicates a fast exchange system.

We then tested the applicability of the ^19^F NMR method to monitor binding of weak inhibitors, exemplified by enantiomeric isoquinolines **3** (Figure 3 B) and **4**[20] for which structural information is unavailable. Upon titration of NDM-1* with isoquinoline **3** a change in the chemical shift (Δ*δ*_max_=0.13 ppm) and slight line broadening was observed, likely corresponding to an average of both non-ligand-bound NDM-1* and NDM-1*-**3** complex signals (Figure 3 B), that is, characteristic of a fast exchange systems (contrasting with the slow exchange system observed for l- and D-captopril). With **4** significant line broadening upon complex formation was observed, which is typical for an intermediate exchange system (Figure S12). These results are in agreement with the reported IC_50_ data; that is, **4** is a better inhibitor than **3** (IC_50_ 61 μM for **3** and 47 μM for **4**).[20] Displacement of the weaker binding ligand **4** by a stronger binder (D-captopril) led to the formation of NDM-1*-D-captopril complex (Figure S14). The data obtained for isoquinoline **3** allowed for binding isotherm fitting using the Equation (1) defining the dependent variable as Δ_obs_/Δ_max_ (where where Δ_obs_=*δ*_0_−*δ*_obs_, Δ_max_=*δ*_0_−*δ*_max_ and *δ*_0_, *δ*_obs_, *δ*_max_ are chemical shifts of the initial protein peak, observed peak and peak with maximal shift corresponding to a saturated protein–ligand complex, respectively), to give a *K*_D_ of 131 μM (Figure S15).

Monobactams, for example, aztreonam, are not hydrolyzed by MBLs[21] and are reported not to inhibit NDM-1,[9b] however the Bc-II MBL binds aztreonam in a non-productive mode, as shown by NMR spectroscopy.[21] However, no ^19^F chemical shift change was observed upon the addition of aztreonam (2.5 mM) to NDM-1 suggesting aztreonam does not bind to NDM-1, consistent with the lack of observed inhibition.

In conclusion we have developed a robust and efficient ^19^F NMR method for monitoring conformational changes and specific ligand binding to NDM-1. The labeling method is efficient and complementary to genetic incorporation methods. The technique enables determination of binding constants, assignment of the type and number of ligand binding modes. In combination with conventional screening methods, the method should aid in the identification of MBL inhibitors.

## References

[1a] Cornaglia G, Giamarellou H, Rossolini GM (2011). Lancet Infect. Dis.

[1b] Walsh TR, Toleman MA, Poirel L, Nordmann P (2005). Clin. Microbiol. Rev.

[1c] Bebrone C (2007). Biochem. Pharmacol.

[2] Fisher JF, Meroueh SO, Mobashery S (2005). Chem. Rev.

[3a] McKenna M (2013). Nature.

[3b] O’Connell KMG, Hodgkinson JT, Sore HF, Welch M, Salmond GPC, Spring DR (2013). Angew. Chem.

[3c] Spencer J, Walsh TR (2006). Angew. Chem.

[4] Bush K, Jacoby GA (2010). Antimicrob. Agents Chemother.

[5] Moali C, Anne C, Lamotte-Brasseur J, Groslambert S, Devreese B, Van Beeumen J, Galleni M, Frere JM (2003). Chem. Biol.

[6] Kumarasamy KK, Toleman MA, Walsh TR, Bagaria J, Butt F, Balakrishnan R, Chaudhary U, Doumith M, Giske CG, Irfan S, Krishnan P, Kumar AV, Maharjan S, Mushtaq S, Noorie T, Paterson DL, Pearson A, Perry C, Pike R, Rao B, Ray U, Sarma JB, Sharma M, Sheridan E, Thirunarayan MA, Turton J, Upadhyay S, Warner M, Welfare W, Livermore DM, Woodford N (2010). Lancet Infect. Dis.

[7a] Chen H, Viel S, Ziarelli F, Peng L (2013). Chem. Soc. Rev.

[7b] Cobb SL, Murphy CD (2009). J. Fluorine Chem.

[7c] Danielson MA, Falke JJ (1996). Annu. Rev. Biophys. Biomol. Struct.

[7d] Vulpetti A, Dalvit C (2012). Drug Discovery Today.

[8a] Gerig JT (1994). Prog. Nucl. Magn. Reson. Spectrosc.

[8b] Kitevski-LeBlanc JL, Prosser RS (2012). Prog. Nucl. Magn. Reson. Spectrosc.

[9a] Kim Y, Cunningham MA, Mire J, Tesar C, Sacchettini J, Joachimiak A (2013). FASEB J.

[9b] Kim Y, Tesar C, Mire J, Jedrzejczak R, Binkowski A, Babnigg G, Sacchettini J, Joachimiak A (2011). PloS One.

[9c] Zhang H, Hao Q (2011). FASEB J.

[10] Brown WE, Seamon KB (1978). Anal. Biochem.

[11] King DT, Worrall LJ, Gruninger R, Strynadka NCJ (2012). J. Am. Chem. Soc.

[12a] Huestis WH, Raftery MA (1978). Biochem. Biophys. Res. Commun.

[12b] Thomas MR, Boxer SG (2001). Biochemistry.

[13] Bain AD (2003). Prog. Nucl. Magn. Reson. Spectrosc.

[14] Kitevski-LeBlanc JL, Evanics F, Prosser RS (2009). J. Biomol. NMR.

[15] Guo Y, Wang J, Niu G, Shui W, Sun Y, Zhou H, Zhang Y, Yang C, Lou Z, Rao Z (2011). Protein Cell.

[16] Morton CJ, Pugh DJR, Brown ELJ, Kahmann JD, Renzoni DAC, Campbell ID (1996). Structure.

[17] Antony J, Gresh N, Olsen L, Hemmingsen L, Schofield CJ, Bauer R (2002). J. Comput. Chem.

[18] García-Sáez I, Hopkins J, Papamicael C, Franceschini N, Amicosante G, Rossolini GM, Galleni M, Frère J-M, Dideberg O (2003). J. Biol. Chem.

[19] Liénard BMR, Garau G, Horsfall L, Karsisiotis AI, Damblon C, Lassaux P, Papamicael C, Roberts GCK, Galleni M, Dideberg O, Frère JM, Schofield CJ (2008). Org. Biomol. Chem.

[20] van Berkel SS, Brem J, Rydzik AM, Salimraj R, Cain R, Verma A, Owens RJ, Fishwick CW, Spencer J, Schofield CJ (2013). J. Med. Chem.

[21] Poeylaut-Palena AA, Tomatis PE, Karsisiotis AI, Damblon C, Mata EG, Vila AJ (2007). Bioorg. Med. Chem. Lett.

