# Supplementary material for: Monitoring Conformational Changes in the NDM-1 Metallo-β-lactamase by 19F NMR Spectroscopy
Source: Angew Chem Int Ed Engl. 2014 Feb 24;53(12):3129–33. doi: 10.1002/anie.201310866 (PMC4499255; doi:10.1002/anie.201310866)
Supplement: Supplementary file 1 [file anie0053-3129-sd1.pdf]

Angewandte Chemie
Eine Zeitschrift der Gesellschaft Deutscher Chemiker

Supporting Information
© Wiley-VCH 2014
69451 Weinheim, Germany

# Monitoring Conformational Changes in the NDM-1 Metallo-β-lactamase by $^{19}$F NMR Spectroscopy**

*Anna M. Rydzik, Jürgen Brem, Sander S. van Berkel, Inga Pfeffer, Anne Makena, Timothy D. W. Claridge,* and Christopher J. Schofield**

anie_201310866_sm_miscellaneous_information.pdf

# Table of Contents

General Experimental .......... 2

Materials .......... 2

Mutagenesis .......... 2

Protein production and purification .......... 2

NDM-1 M67C labelling .......... 3

Kinetic analyses .......... 3

NMR experiments .......... 3

Metal binding experiments .......... 3

Figure S1. Overall fold of NDM-1-2Zn(II) complex. .......... 4

Figure S2. Overlay of views derived from NDM-1 crystal structures. .......... 5

Figure S3. Purification of NDM-1 M67C. .......... 6

Figure S4. MS spectra of NDM-1 M67C and NDM-1 M67C labelled with BFA. .......... 7

Tryptic digest and MS/MS analysis .......... 8

In-Solution Digestion .......... 8

MALDI-ToF-MS and MS/MS studies .......... 8

Figure S5a. MALDI-ToF MS spectra of labelled and unlabelled M67C NDM-1 digested with trypsin. .......... 9

Figure S5b. MALDI-ToF-ToF fragmentation of a labelled precursor .......... 10

Figure S5c. MALDI-ToF-ToF fragmentation of unlabelled precursor .......... 11

Figure S6 Meropenem and nitrocefin turnover by NDM-1 and NDM-1*. .......... 12

Figure S7. Dose-response curves for the inhibition of NDM-1 and NDM-1* by L- and D-captopril. .......... 12

Figure S8. Solvent exposure analyses using $^{19}F$ NMR. .......... 12

Figure S9. Time course analyses of NDM-1* treatment with EDTA followed by $^{19}F$ NMR. .......... 13

Figure S10. Binding of D- and L-captopril to NDM-1* as followed by $^{19}F$ NMR. .......... 13

Figure S11. $K_D$ measurements using $^{19}F$ NMR. .......... 14

Figure S12. Titration of di-Zn(II)-NDM-1* with isoquinoline (4). .......... 14

Figure S13. DMSO induced chemical shift experiments. .......... 15

Figure S14 $^{19}F$ NMR spectra of NDM-1* treated with isoquinoline (4) .......... 15

Figure S15. Formation of the di-Zn(II)-NDM-1* complex with (3) as observed by $^{19}F$ NMR. .......... 16

References .......... 16

# General Experimental

## Materials

Chemicals were from Sigma-Aldrich. Isoquinolines (**3**) ((*S*)-2-(1-chloro-4-hydroxyisoquinoline-3-carboxamido)-3-(1H-indol-3-yl)propanoic acid) and (**4**) ((*R*)-2-(1-chloro-4-hydroxyisoquinoline-3-carboxamido)-3-(1H-indol-3-yl)propanoic acid) were synthesized according to the reported procedure[1]. Thiol (**2**) was synthesized as described[2].

## Mutagenesis

The M67C NDM-1 variant was generated using the δ36 NDM-1 in the pOPINF plasmid as described[1]. The desired point mutation was achieved employing the QuikChange sites directed mutagenesis kit (Stratagene) using the following primers:
forward – 5ˈ- GGCAGCATACCAGCTATCTGGATTGCCCTGGTTTTGGTGCAG,
reverse – 5ˈ- CTGCACCAAAACCAGGGCAATCCAGATAGCTGGTATGCTGCC.

## Optimisation of protein expression system

In order to obtain a high-yield protein expression system to produce the NDM-1 M67C mutant first the same expression system was applied that we used to produce the wild type NDM-1.[ref] (Namely/In details: The recombinant NDM-1 were produced in *E. coli* BL21(DE3) pLysS cells using 2TY media supplemented with 50 μg/ml ampicillin and 34 μg/ml chloramphenicol. Cells were grown at 37 °C until the $OD_{600}$ reached 0.6 - 0.7. After that the temperature was lowered to 20 °C and the cells were induced with 0.5 mM IPTG. The cells were further incubated for 20h before harvested). Using the wild type NDM-1 expression system low expression of the NDM-1 M67C mutant was obtained (< 0.5 mg/L of culture), which was not suitable for us and therefore we decided to optimize the expression system. During the optimization process the expression level was judged by the SDS page that contained the lysate of the cells obtained from the lysis with the BugBuster kit.©

To improve the expression level we tried to use different temperatures (37, 28 and 18 ºC), various IPTG levels (0, 0.1, 0.25, 0.5 and 1mM final IPTG) and different incubation times (4 and 20h), but in all cases low expression levels were obtained. We also tried to use normal BL21(DE3) cells, made the IPTG induction at higher OD (~ 1.3) or made the expression of the NDM-1 M67C mutant in the presence of 50 μg/ml $ZnSO_4$, but the expression level was not significantly improved. Next, we also tried to use other media such as the Terrific Broth (TB media, using a 0.5 mM IPTG) and the Studier autoinduction media to improve the expression level. During the expression trials using TB and autoinduction media we also tried different temperatures (37, 28 and 18 ºC). A significantly improved level of expression was obtained by using the autoinduction media at 18 ºC.

## Protein production and purification

Recombinant NDM-1 M67C was produced in *E. coli* BL21 (DE3) pLysS cells using Studier autoinduction media[3] supplemented with 50 μg/ml ampicillin and 50 μg/ml chloramphenicol. The medium was modified by addition of $MgSO_4$ and $ZnSO_4$, to ensure the protein was obtained as di-Zn(II) complex (the Studier autoinduction media contains various metals salts and using original recipe different metal-NDM-1 complexes were obtained). Cells were grown at 37 ºC for 4 h, then the temperature was reduced to 18 ºC for 20h. Cell were harvested by centrifugation (10 min, 8000 rpm) and NDM-1 M67C was purified as reported[1]. FPLC chromatograms and SDS-page gels from the NDM-1 M67C purification are shown in Fig. S3.

**NDM-1 M67C labelling**

NDM-1 M67C was treated with tris-(2-carboxyethyl)phosphine (TCEP, final concentration 2 mM), then incubated on ice (5 min) and buffered exchanged into phosphate buffer (50 mM, pH 7.0, 200 mM NaCl). A concentrated stock of 100 mM 3-bromo-1,1,1-trifluoroacetone reagent in phosphate buffer was prepared freshly prior to the reaction. NDM-1 M67C sample (0.15 mM) was treated with 3-bromo-1,1,1-trifluoroacetone (final concentration 2 mM) and incubated for 10 min at room temperature, prior to buffer exchange into Tris buffer (50 mM, pH 7.5, 200 mM NaCl). Buffer exchanges were performed using a PD-10 desalting column (GE Healthcare).

**Kinetic analyses**

Kinetic and inhibition analyses were performed as described[1]. $K_M$ measurements were performed using 0.5 nM NDM-1 and 1 nM NDM-1* for meropenem measurements and 0.25 nM NDM-1 and 2 nM NDM-1* for nitrocefin measurements.

**NMR experiments**

NMR measurements were conducted using a Bruker AVII 500 spectrometer equipped with a 5mm z-gradient triple resonance inverse $^{1}H/^{19}F(^{13}C)$ TXI probe operating at 298K using 5 mm diameter NMR tubes (Norell). $^{19}F\{^{1}H\}$ decoupled spectra were recorded using inverse-gated Waltz-16 $^{1}H$ decoupling with rf field of 3.125 kHz. $^{19}F$ 90$^{o}$ pulse lengths were 11 µs and spectra were typically obtained using 768 scans and a recovery delay of 1s. Data were processed with 3 Hz Lorentzian line broadening using TopSpin 3.1 software (Bruker) and were referenced to the internal TFA standard (-75.45 ppm).

Samples contained the NDM-1*-2Zn(II) complex (80 µM, unless otherwise stated) in Tris buffer (50 mM, pH 7.5) supplemented with 200 mM NaCl and 10% $D_2O$. Trifluoroacetic acid (TFA, 50 µM) was used as an internal standard. All inhibitor incubations were compared to appropriate NDM-1* samples containing same amount of DMSO, but no inhibitor, to ensure change of chemical shift was not the sole effect of increasing DMSO concentration. A DMSO titration curve was used for reference (Fig. S11).

**Metal binding experiments**

Apo NDM-1* was obtained by incubation of the NDM-1*-2Zn(II) complex with 10 mM EDTA in Tris buffer (50 mM pH 7.5) supplemented with 200 mM NaCl (12 h, 4°C). The solution was then buffer exchanged into fresh Tris buffer (50 mM pH 7.5) supplemented with 200 mM NaCl to remove excess EDTA. Samples for metal titrations were containing 70 µM NDM-1* and 50 µM TFA as an internal standard.

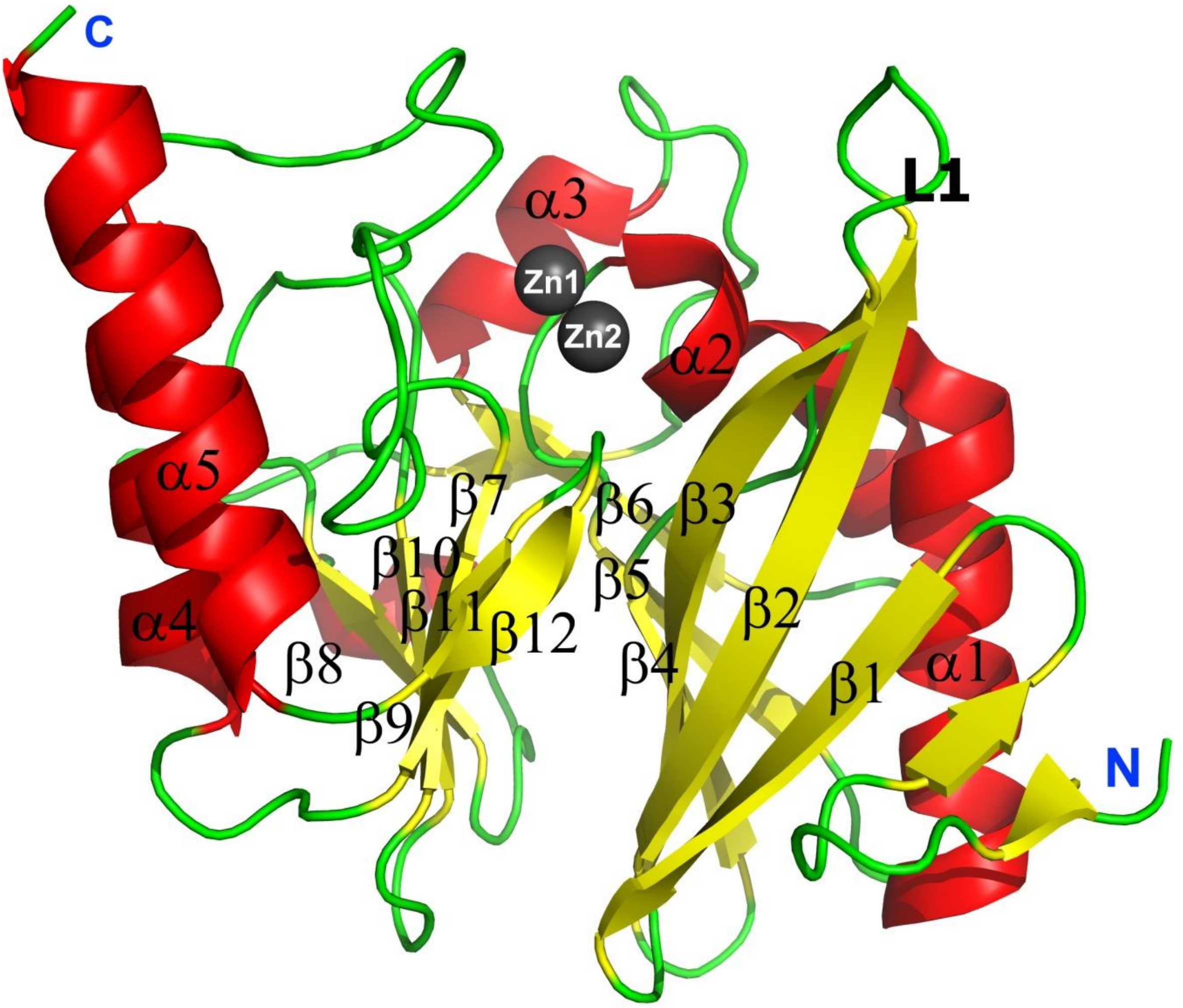


**Figure S1. Overall fold of NDM-1-2Zn(II) complex.** Note the L1 loop, which was labelled in our studies, flanks the NDM-1 active site. The figure was generated using a di-Zn(II)-NDM-1 complex crystal structure (PDB id: 3SPU)[4].

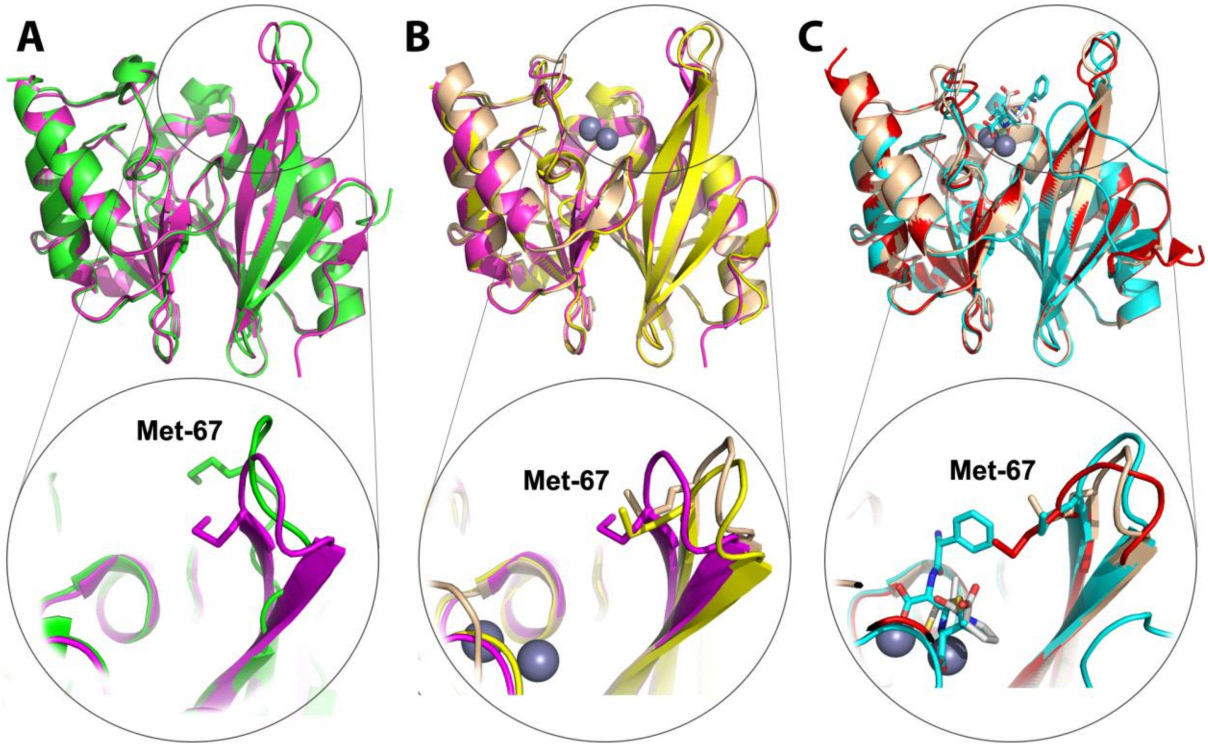


**Figure S2. Overlay of views derived from NDM-1 crystal structures.** The L1 loop (circled) can adopt different conformations dependent on the metallation state or the ligands bound. A – Comparison of views from two different apo NDM-1 structures 3RKK[5] (green) and 3SBL[5] (magenta) implies conformational flexibility of the L1 loop in the apo-protein. B – Comparison of apo NDM-1 (3SBL, magenta), mono-Zn(II) NDM-1 complex (3SFP[5], yellow) and di-Zn(II) NDM-1 complex (3SPU, wheat) views indicates that the L1 loop can adopt different conformations reflecting different metallation states. C – Comparison of structures for the NDM-1-2Zn(II) complex (3SPU[4], wheat), the NDM-1-2Zn(II) in complex with hydrolysed ampicillin (3Q6X[6], blue), and the NDM-1-2Zn(II) in complex with L-captopril (4EXS[7], red) implies the L1 loop can adopt different conformation depending on the ligand bound. The close-ups show the different positions of the chain Met67 side on the L1 loop. The labelled loop (L1) has average B-factor ~59 Å$^2$ versus the ~28 Å$^2$ for the whole enzyme that supports the conformational flexibility of the loop (based on the crystal structure of NDM-1 with PBD code: 3SPU).

**A**

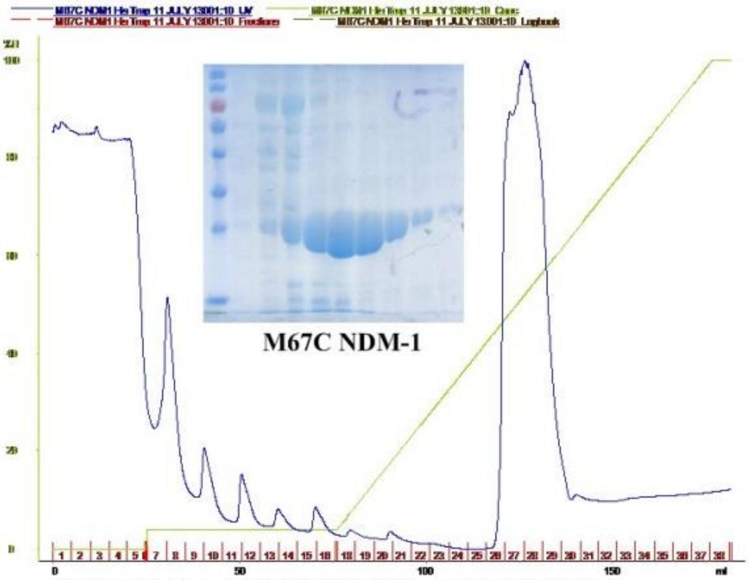


**B**

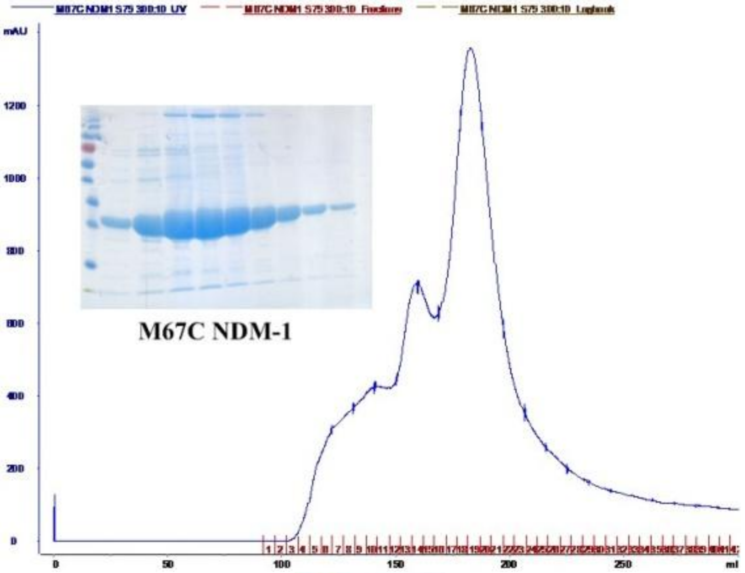


**C**

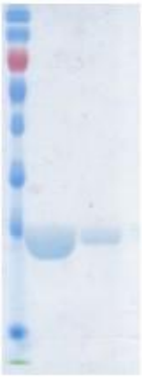

**Figure S3. Purification of NDM-1 M67C.** FPLC traces of NDM-1 M67C purification by: A – HisTrap HP column (Ni-NTA resin) and B – gel filtration column (Superdex S200). C – SDS-PAGE gel of NDM-1 M67C after His-tag cleavage (lane 2 and 3). Lane 1- molecular weight markers (PageRuler Prestained Protein Ladder 10-170 kDa, Thermo Scientific).

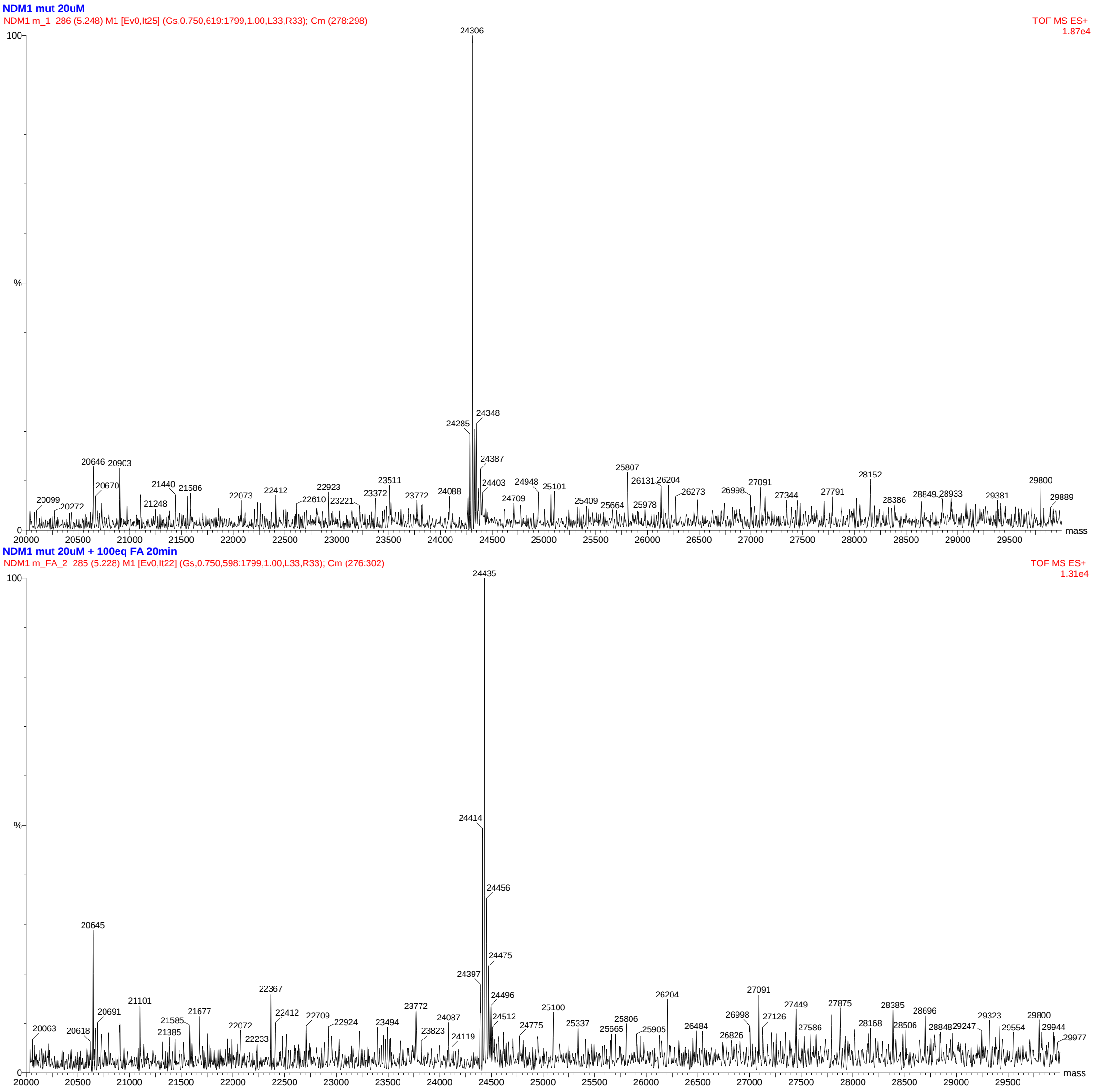


**Figure S4. MS spectra of NDM-1 M67C (top) and NDM-1 M67C labelled with BFA (3-bromo-1,1,1-trifluoro acetone) (bottom).** The observed mass difference (+129) corresponds to attachment of a single BFA label per a NDM-1 M67C molecule. Spectra were acquired using a Waters LCT Premier instrument fitted with TOF analyser. The electrospray ionisation mode was used.

**Tryptic digest and MS/MS analysis**

**In-Solution Digestion**

All reagents were prepared in 100 mM Tris buffer, pH 7.8. Samples (< 500 µg) were dried in a Vacufuge® vacuum concentrator (*Eppendorf*) connected to an external diaphragm pump and then resuspended in 100 µl of 6 M urea. Disulfides were reduced at room temperature (30 min) by addition of 5 µl of 200 mM dithiothreitol (DTT), and subsequently alkylated at room temperature (30 min) with 30 µl of 200 mM iodoacetamide. Unreacted alkylating reagent was consumed by addition of 30 µl DTT solution (30 min). The sample was then diluted with 775 µl Tris buffer, mixed with Sequencing Grade Modified Porcine Trypsin (*Promega*) at trypsin:protein sample ratio of ca. 1:50, and digested at 37°C for 12h. Digestion was stopped by adjusting the sample to pH 3-4 by addition of concentrated acetic acid. Digested samples were subsequently purified and desalted by solid-phase extraction using Sep-Pak $C_{18}$ Plus Light Cartridges (*Waters*, 130 mg sorbent per cartridge, 55-105 µM particle size) following the manufacturer's protocol. The samples were then dried using a Vacufuge® and subsequently re-dissolved in typically 15-30 µl of 50% $CH_3CN$/0.1% $CF_3COOH$ for analysis by MALDI-ToF-MS.

**MALDI-ToF-MS and MS/MS studies**

Matrix-assisted laser desorption/ionization time of flight mass spectrometry (MALDI-ToF-MS) and MS/MS analyses were performed using a *Bruker Daltonics* Ultraflex™ MALDI-ToF/ToF machine, using flexControl™ 3.0 software. Spectra were recorded in the positive ion reflectron mode, typically with 32-38% laser energy. Calibration was performed on each day prior to the measurements using Peptide Calibration Standard II (*Bruker Daltonics*). Data were processed using *Bruker Daltonics* flexAnalysis™ 3.0 software and assigned manually. For MALDI measurements, 1 µl of the sample was mixed with 4 µl of 2,5-dihydroxybenzoic acid (DHB, 20 mg/ml in 50% $CH_3CN$/0.1% $CF_3COOH$) matrix and 2 µl of this sample-matrix mixture spotted onto a 24 x 16 MTP AnchorChip™ 384 T F MALDI target and allowed to air-dry before analysis.

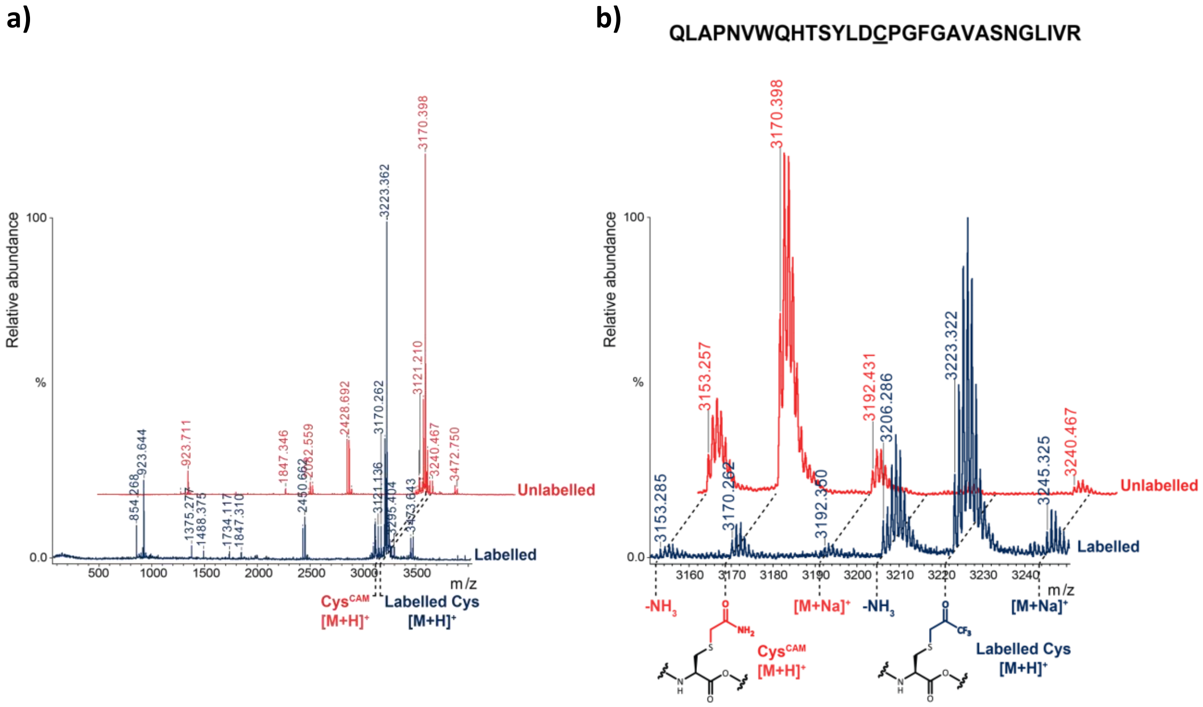


**Figure S5a. MALDI-ToF MS spectra of labelled and unlabelled M67C NDM-1 digested with trypsin.** Cysteine residues were reduced (DTT) and *S*-carbamidomethylated (Cys$^{CAM}$) prior to digestion. a) Complete 'spectrum' of the digested sample. b) Close-up view of the assigned modified peptide (labelled: m/z = 3223.3, *S*-carbamidomethylated: m/z 3170.4).

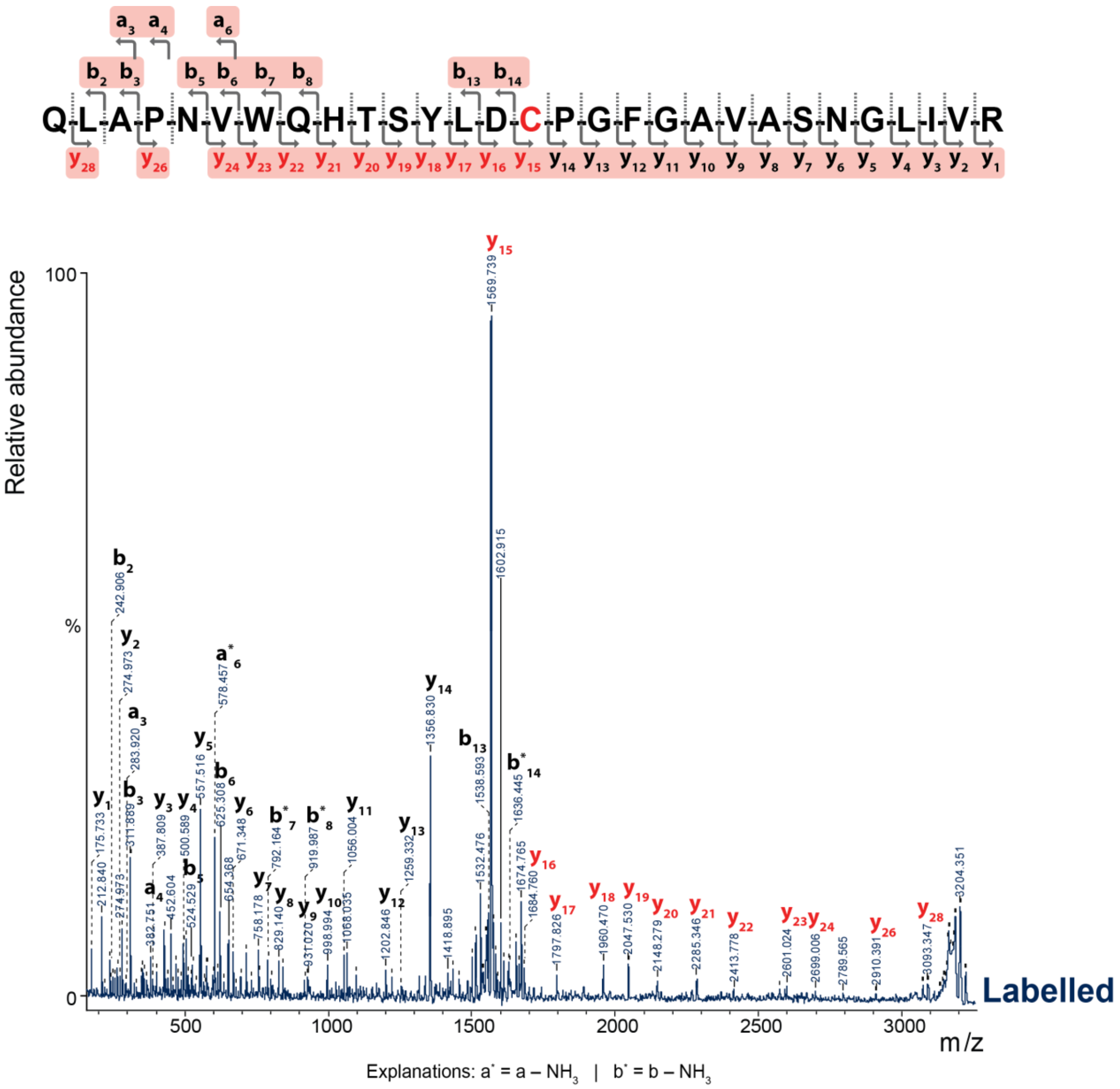


**Figure S5b. MALDI-ToF-ToF fragmentation of a labelled precursor** [QLAPNVWQHTSYLD<u>**C**</u>PGFGAVASNGLIVR+H]$^+$ (m/z = 3223.3) from trypsin digestion of the BFA-labelled NDM-1 mutant M67C. The data support modification at C67. Ions coloured in red have the label (+110 Da, relative to unmodified cysteine, or +54 Da relative to *S*-carbamidomethylated cysteine in the unlabelled control respectively), black coloured ions are unlabelled.

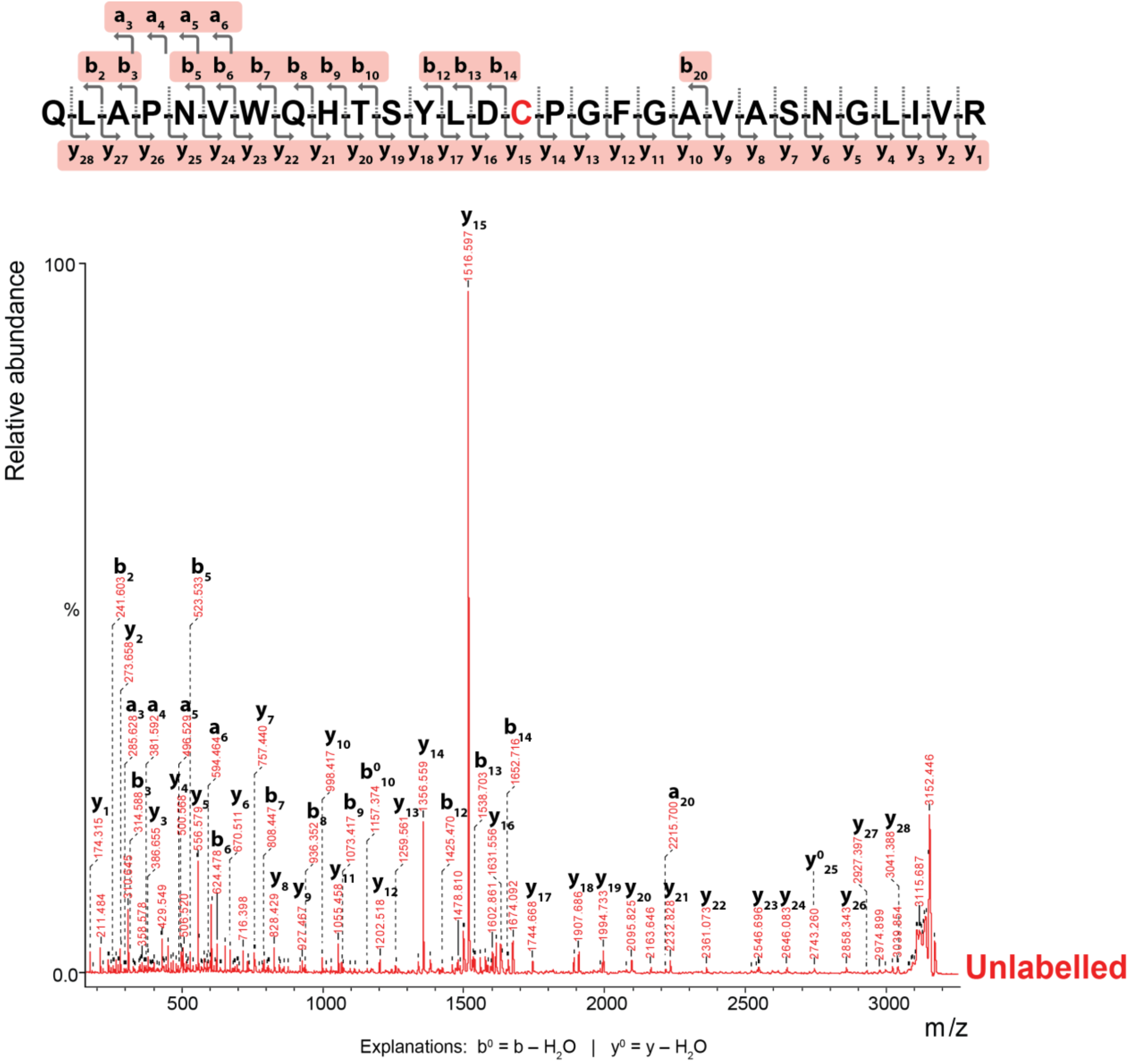


**Figure S5c. MALDI-ToF-ToF fragmentation of unlabelled precursor** [QLAPNVWQHTSYLDCPGFGAVASNGLIVR+H]$^+$ (m/z = 3170.4) from a tryptic digestion of unlabelled M67C NDM-1 with *S*-carbamidomethylated C67 as a control.

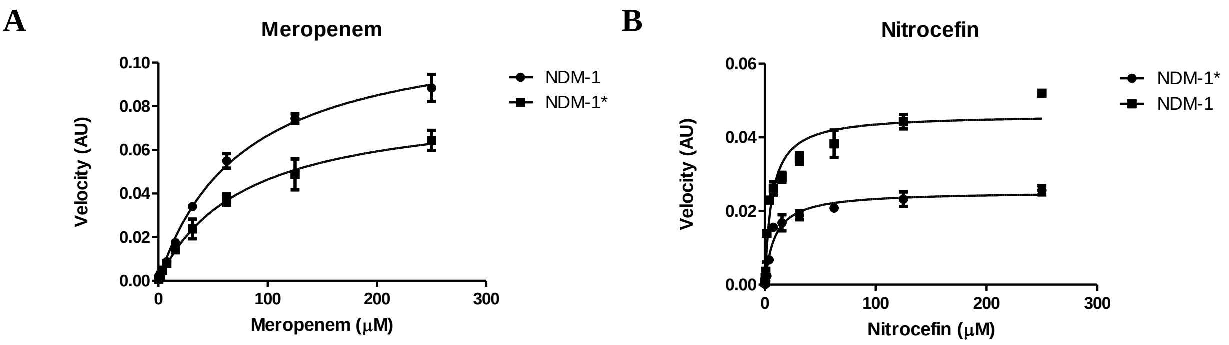


**Figure S6 Meropenem and nitrocefin turnover by NDM-1 and NDM-1*.**

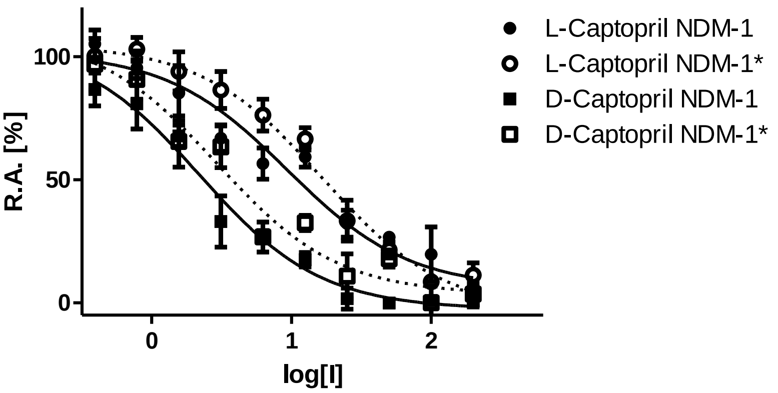


**Figure S7. Dose-response curves for the inhibition of NDM-1 and NDM-1* by L- and D-captopril.**

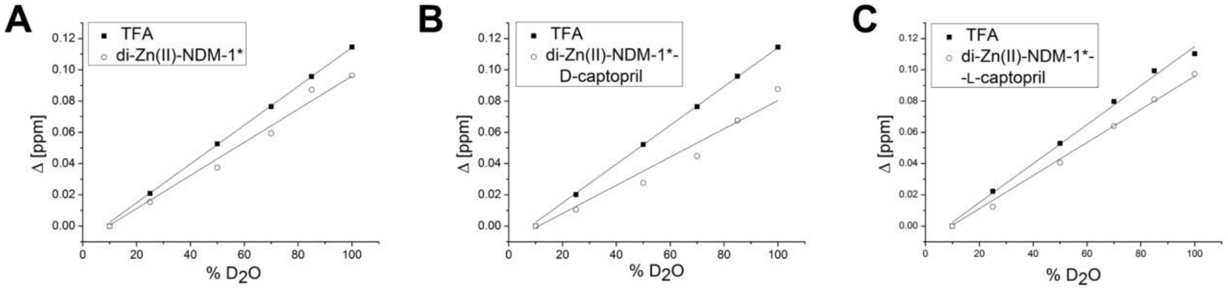


**Figure S8. Solvent exposure analyses using $^{19}$F NMR.**

Monitoring the change in $^{19}$F chemical shift as a function of $D_2O$ content. A – Change in chemical shift for $CF_3COOH$ (TFA) (■) and di-Zn(II)-NDM-1* (○), fitted with linear functions giving (■) y=0.00124x-0.01, $R^2$>0.99 and (○) y=0.00106x-0.01, $R^2$>0.98. B – Change in chemical shift for TFA (■) and the di-Zn(II)-NDM-1*-D-captopril complex (○), fitted with linear functions giving (■) y=0.00124x-0.01, $R^2$>0.99 and (○) y=0.00090x-0.01, $R^2$>0.98. C – Change in chemical shift for TFA (■) and the di-Zn(II)-NDM-1*-L-captopril complex (○), fitted with linear functions giving (■) y=0.00125x-0.01, $R^2$>0.99 and (○) y=0.00106x-0.01, $R^2$>0.99. The percentage solvent exposures of NDM-1* label were calculated as a ratio of the slope obtained for the NDM-1* complex relative to the analogous slope for TFA in the sample (set to 100% as a fully exposed small molecule signal).

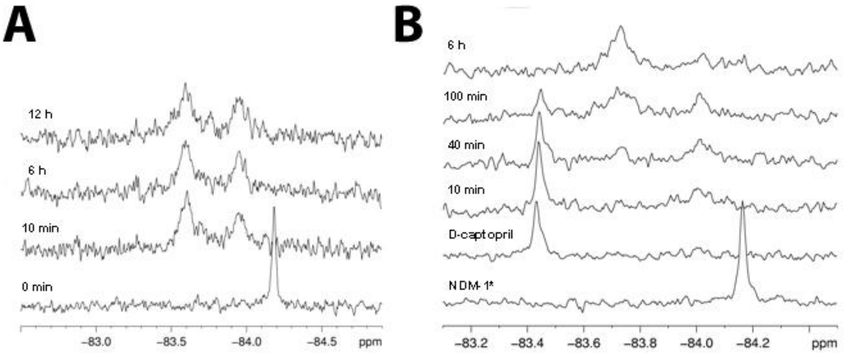


**Figure S9. Time course analyses of NDM-1* treatment with EDTA followed by $^{19}$F NMR.** A – Sequestering metal from the NDM-1*-2Zn(II) complex by 10 mM EDTA is rapid. B – Treatment of the NDM-1*-2Zn(II)-D-captopril complex with 10 mM EDTA leads to a decrease in the signal corresponding to the initial complex and the appearance of the peaks characteristic of apo protein.

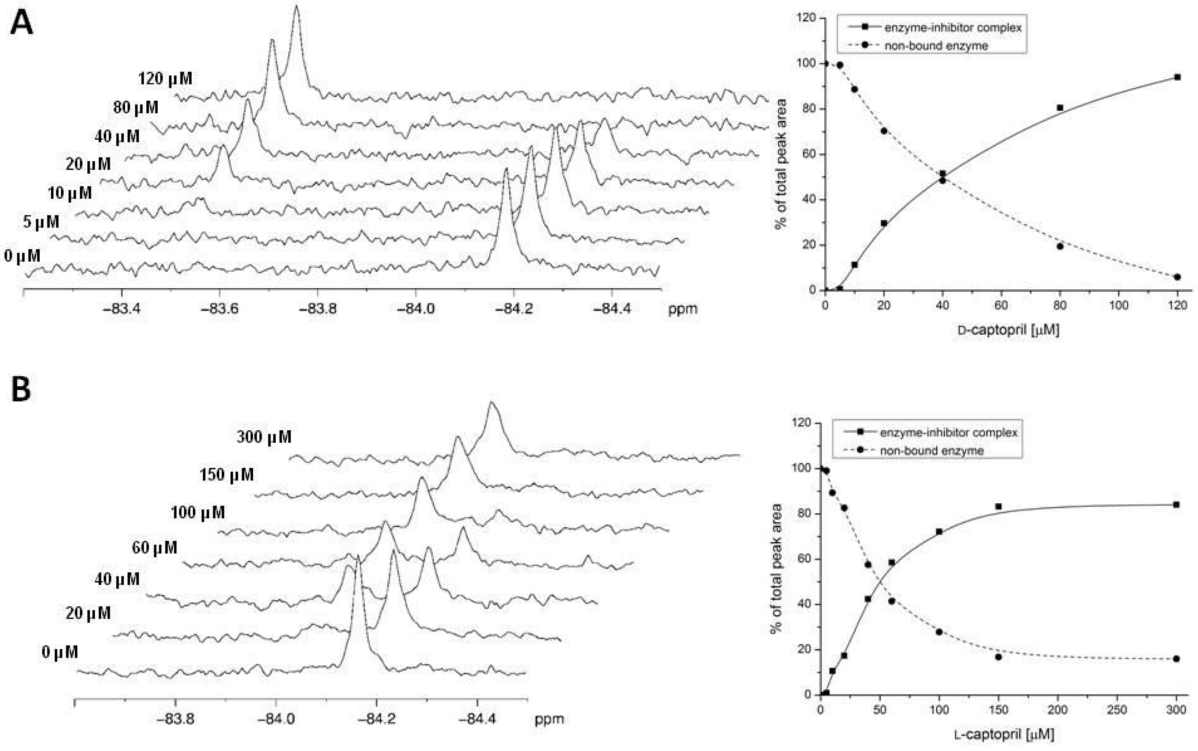


**Figure S10. Binding of D- (A) and L-captopril (B) to NDM-1* as followed by $^{19}$F NMR.**
Conditions were as described in the Experimental Section.

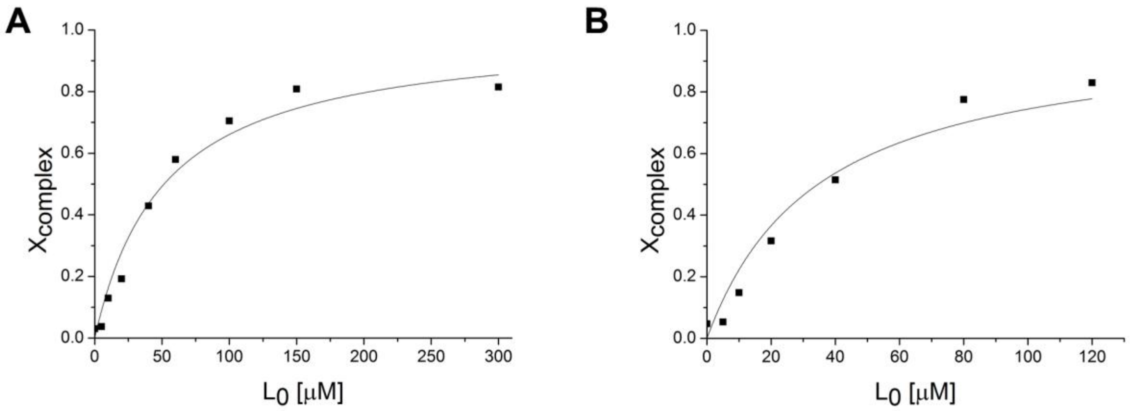


**Figure S11. $K_D$ measurements using $^{19}$F NMR.**

Molar fraction of protein-ligand complex (L- captopril (A) and D -captopril (B)) as a function of free ligand concentration. Data were fitted with equation (**1**).

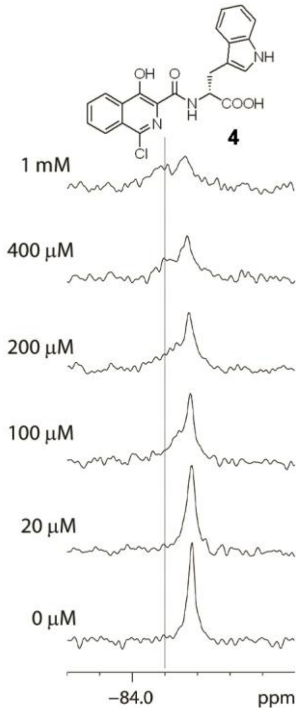


**Figure S12. Titration of di-Zn(II)-NDM-1* (70 μM) with isoquinoline (4).** Significant signal broadening and appearance of a second peak is observed at higher concentrations of isoquinoline (**4**). The change into a $^{19}$F chemical shift of the original di-Zn(II)-NDM-1* peak is attributed to increasing DMSO concentration in the sample, as determined by titration with DMSO (Figure S13). Note the binding mode of isoquinoline inhibitor of MBLs is unknown.

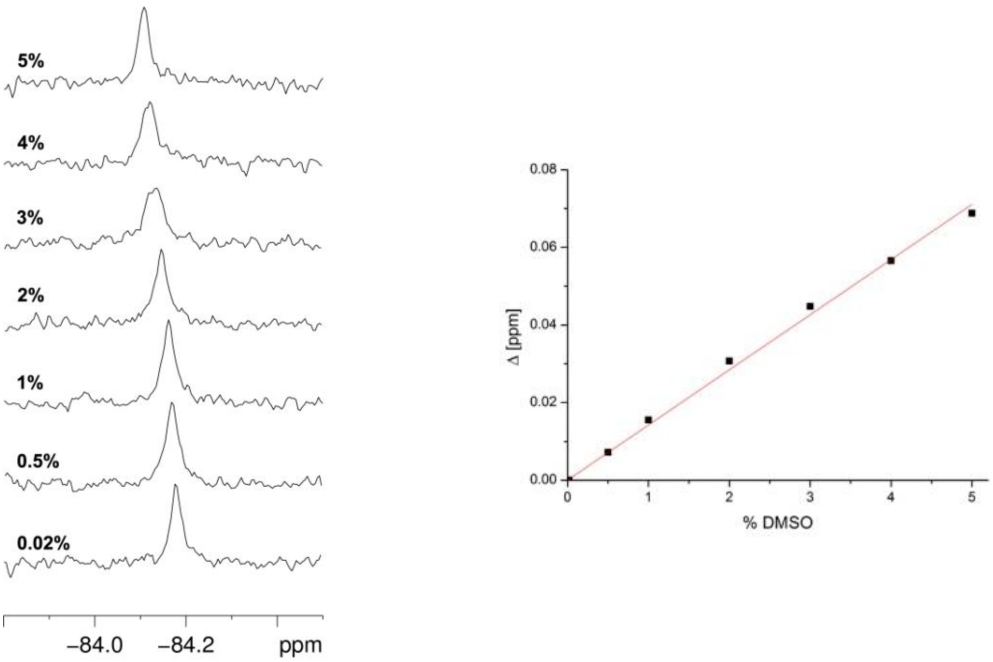


**Figure S13. DMSO induced chemical shift experiments.** Increasing the concentration of DMSO induces a change in chemical shift of the $^{19}F$ signal. When the change in the $^{19}F$ $CF_3$-COOH signal shift is subtracted from the corresponding change in the NDM-1* chemical shift, a DMSO induced change in signal shift is apparent. The dependence of signal shift on DMSO content can be fitted with a linear function as follows: y=0.01421x, $R^2$>0.99 (OriginPro 8.5.1).

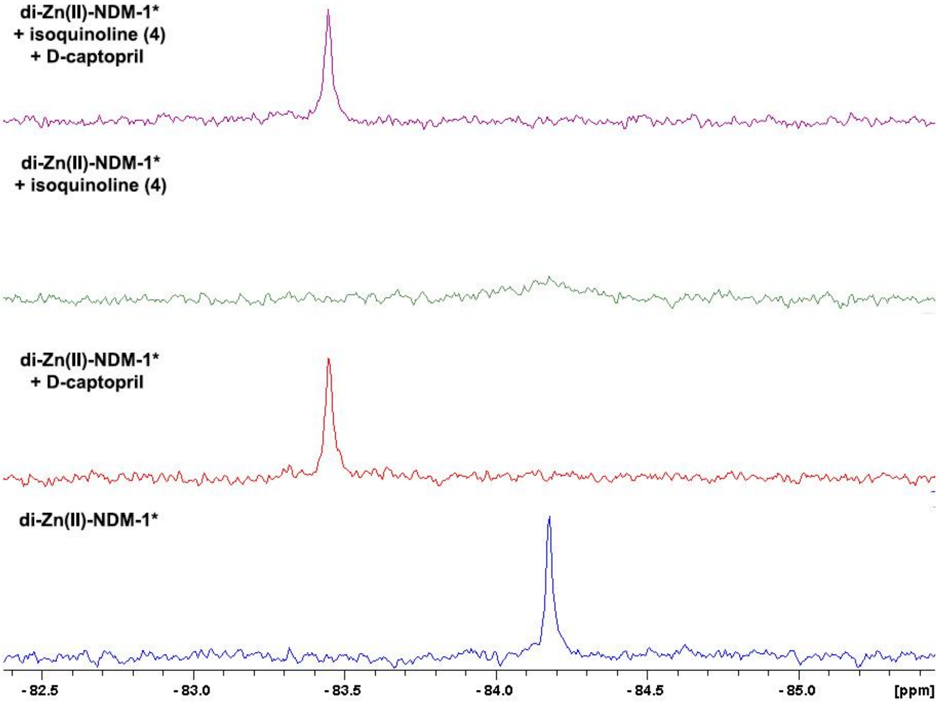


**Figure S14 $^{19}F$ NMR spectra of NDM-1* treated with isoquinoline (4) then D-captopril.** Note the $^{19}F$ signal recovers after addition of D-captopril.

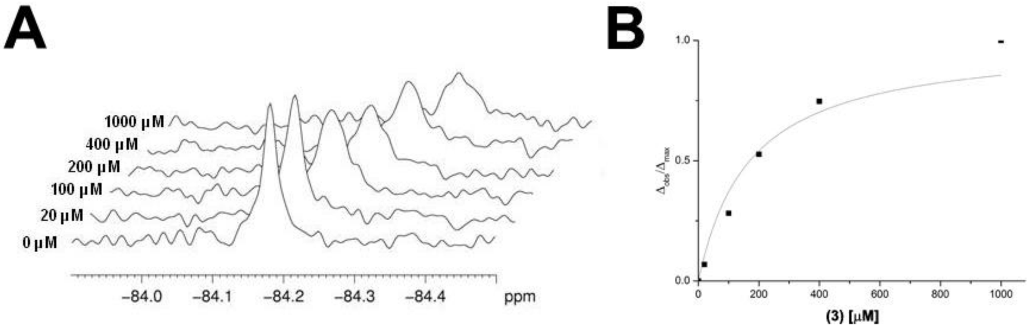


**Figure S15. Formation of the di-Zn(II)-NDM-1* complex with (3) as observed by $^{19}$F NMR.**

A – $^{19}$F NMR spectra of titration of (**3**) into a di-Zn(II)-NDM-1* solution. B – $K_D$ fitting for binding of (**3**) to di-Zn(II)-NDM-1* gives a value 131±24 µM. Data were fitted with equation (1) (see main text, OriginPro 8.5.1) defining the dependent variable as $\Delta_{obs}/\Delta_{max}$ , where $\Delta_{obs} = \delta_0 - \delta_{obs}$, $\Delta_{max} = \delta_0 - \delta_{max}$ and $\delta_0$, $\delta_{obs}$, $\delta_{max}$ are chemical shifts of the initial protein peak, observed peak and peak with maximal shift corresponding to saturated protein-ligand complex, respectively.

**References**

(1) van Berkel, S. S.; Brem, J.; Rydzik, A. M.; Salimraj, R.; Cain, R.; Verma, A.; Owens, R. J.; Fishwick, C. W.; Spencer, J.; Schofield, C. J. *J. Med. Chem.* **2013**, *56*, 6945.
(2) Liénard, B. M. R.; Garau, G.; Horsfall, L.; Karsisiotis, A. I.; Damblon, C.; Lassaux, P.; Papamicael, C.; Roberts, G. C. K.; Galleni, M.; Dideberg, O.; Frère, J. M.; Schofield, C. J. *Org. Biomol. Chem.* **2008**, *6*, 2282.
(3) Studier, F. W. *Protein Expres. Purif.* **2005**, *41*, 207.
(4) King, D.; Strynadka, N. *Protein Sci.* **2011**, *20*, 1484.
(5) Kim, Y.; Tesar, C.; Mire, J.; Jedrzejczak, R.; Binkowski, A.; Babnigg, G.; Sacchettini, J.; Joachimiak, A. *PloS one* **2011**, *6*, e24621.
(6) Zhang, H.; Hao, Q. *FASEB J.* **2011**, *25*, 2574.
(7) King, D. T.; Worrall, L. J.; Gruninger, R.; Strynadka, N. C. J. *J. Am. Chem. Soc.* **2012**, *134*, 11362.
